# *De novo* transcriptomic analysis of *Chlorella sorokiniana* reveals differential genes expression in photosynthetic carbon fixation and lipid production

**DOI:** 10.1186/s12866-016-0839-8

**Published:** 2016-09-26

**Authors:** Lin Li, Guoqiang Zhang, Qinhong Wang

**Affiliations:** Key Laboratory of Systems Microbial Biotechnology, Tianjin Institute of Industrial Biotechnology, Chinese Academy of Sciences, 32 XiQiDao, Tianjin Airport Economic Area, Tianjin, 300308 People’s Republic of China

**Keywords:** *Chlorella sorokiniana*, Transcriptome, Lipid accumulation, Photosynthetic carbon fixation, RNA-seq

## Abstract

**Background:**

Microalgae, which can absorb carbon dioxide and then transform it into lipid, are promising candidates to produce renewable energy, especially biodiesel. The paucity of genomic information, however, limits the development of genome-based genetic modification to improve lipid production in many microalgae. Here, we describe the *de novo* sequencing, transcriptome assembly, annotation and differential expression analysis for *Chlorella sorokiniana* cultivated in different conditions to reveal the change of genes expression associated with lipid accumulation and photosynthetic carbon fixation.

**Results:**

Six cultivation conditions were selected to cultivate *C. sorokiniana*. Lipid content of *C. sorokiniana* under nitrogen-limited condition was 2.96 times than that under nitrogen-replete condition. When cultivated in light with nitrogen-limited supply, *C. sorokiniana* can use carbon dioxide to accumulate lipid. Then, transcriptome of *C. sorokiniana* was sequenced using Illumina paired-end sequencing technology, and 244,291,069 raw reads with length of 100 bp were produced. After preprocessed, these reads were *de novo* assembled into 63,811 contigs among which 23,528 contigs were found homologous sequences in public databases through Blastx. Gene expression abundance under six conditions were quantified by calculating FPKM value. Ultimately, we found 385 genes at least 2-fold up-regulated while 71 genes at least 2-fold down-regulated in nitrogen-limited condition. Also, 204 genes were at least 2-fold up-regulated in light while 638 genes at least 2-fold down-regulated. Finally, 16 genes were selected to conduct RT-qPCR and 15 genes showed the similar results as those identified by transcriptomic analysis in term of differential expression.

**Conclusions:**

*De novo* transcriptomic analyses have generated enormous information over *C. sorokiniana*, revealing a broad overview of genomic information related to lipid accumulation and photosynthetic carbon fixation. The genes with expression change under different conditions are highly likely the potential targets for genetic modification to improve lipid production and CO_2_ fixation efficiency in oleaginous microalgae.

**Electronic supplementary material:**

The online version of this article (doi:10.1186/s12866-016-0839-8) contains supplementary material, which is available to authorized users.

## Background

The demand of energy is increasing as the world population and global economy continue to grow. Microalgae-based biodiesel, which can realize carbon-neutral by photosynthetic carbon fixation via the microalgae’s growth [1], is a renewable and sustainable energy source. *Chlorella*, one of eukaryotic, unicellular and photosynthetic microorganism, widely distributes in freshwater environment and is capable of accumulating excess lipid in nitrogen-limited condition. Moreover, *Chlorella* were used as a model system for investigating photosynthetic carbon fixation [2, 3]. Due to its various and robust metabolic capacities, *Chlorella* has received increasingly attention as promising microalgae to produce biomass [4], biodiesel [5] as well as high additional-value products [6].

Currently, *Chlorella* is one of the best microalgae as oil feedstock for the production of biodiesel [7]. Particularly, in nitrogen-limited condition, *Chlorella* can alter the metabolic pathways to accumulate a high proportion of lipid which can be used for biodiesel production [8–10]. Although the metabolic transition has been identified in the lipid accumulation process [11], many global changes remain poorly understood, such as genomic information, differential genes expression. As a consequence, the lipid production from naturally occurring *Chlorella* strains is much lower than the theoretical maximum [12], making the cost of biodiesel prohibitively high [13]. One primary cause is the limited understanding of the metabolic pathways in microalgae regulating the lipid metabolism in general and lipid biosynthesis and accumulation in particular [14]. Another cause is the lack of genomic information of some oleaginous but non-model microalgae, which largely hampers the investigation of the transcribed genes and genetic modification in these microalgae to accumulate lipid and other valuable products [15–17].

Transcriptome sequencing could be an efficient and relatively economical method to obtain the functional genomic information without the genomic information [17, 18], providing an initial, broad view of lipid accumulation in nitrogen-limited condition [15] and photosynthetic carbon fixation. A growing number of transcriptomes of oleaginous microalgae were *de novo* sequenced, assembled and annotated to investigate the regulatory mechanism of lipid accumulation [15–18].

In our previous work, we have already identified the metabolic pathways related to lipid accumulation in *C. sorokiniana* based on two transcriptome datasets [19]. In this present study, we sequenced another four transcriptome datasets and analyzed all six transcriptome datasets together to elucidate differential gene expression involved in the lipid accumulation and photosynthetic carbon fixation. In our experiments, the quantity of lipid accumulated under nitrogen-limited condition can be 2.96 times than that under nitrogen-replete condition, making *C. sorokiniana* a promising microalgae strain to produce biodiesel. Then all the six transcriptome datasets were *de novo* assembled, annotated together, and differential genes expression was analyzed as well. Finally, RT-qPCR was conducted for 16 genes involving in the lipid accumulation and photosynthetic carbon fixation. Our results provide an insight into the regulation of lipid metabolism and photosynthetic carbon fixation in *C. sorokiniana* at the transcriptomic level and may contribute to genetic modification in microalgae to improve lipid productivity and carbon fixation efficiency.

## Results and discussion

### Biomass and lipid content under different cultivation conditions

**Fig. 1:**
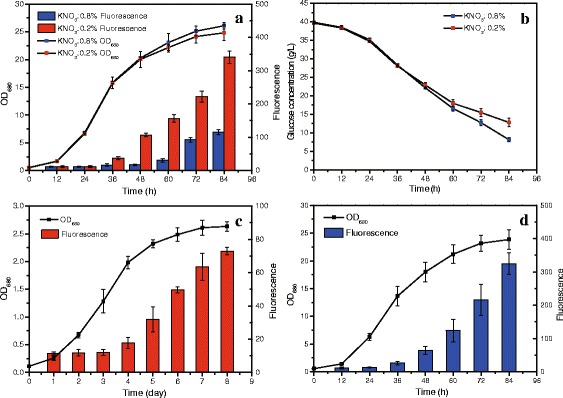
Growth and lipid content of *C. sorokiniana* under different conditions. **a**: Growth and lipid content of *C. sorokiniana* under nitrogen-limited and nitrogen-replete conditions. **b**: The consumption of glucose under nitrogen-limited and nitrogen-replete conditions; **c**: Growth and lipid content of *C. sorokiniana* in light; **d**: Growth and lipid content of *C. sorokiniana* in dark

Six different cultivation conditions were selected to culture *C. sorokiniana* (Table 1), and the growth and lipid content profiles under these conditions were shown in Fig. 1. With 4 % glucose as carbon source, the optical density of the culture at 680 nm (OD_680_) in nitrogen-limited condition (0.2 % KNO_3_ supply) was almost equal to that in nitrogen-replete condition (0.8 % KNO_3_ supply) before 48 h. After 48 h the OD_680_ in nitrogen-replete condition began to become higher than that in nitrogen-limited condition (Fig. 1a). The higher OD_680_ resulted in the more glucose consumption (Fig. 1b) for cell growth, not for lipid production. The nitrogen-limited condition could induce *C. sorokiniana* to accumulate more lipid. After 48 h, the fluorescence intensity of lipid dyed with nile red in nitrogen-limited condition was higher than that in nitrogen-replete condition, meaning that the cells in nitrogen-limited condition accumulated more lipid. At 84 h, The fluorescence intensity in nitrogen-limited condition was as high as 2.96 times than that in nitrogen-replete condition (340 ± 19 and 115 ± 6, respectively Fig. 1a).

**Table 1:** The general information for each sample

	sample A	sample B^a^	sample C	sample D^a^	sample E	sample F
KNO_3_	0.2 %	0.2 %	0.8 %	0.8 %	0.2 %	0.033 %
Carbon source	4 % glucose	4 % glucose	4 % glucose	4 % glucose	4 % glucose	4 % (CO_2_/air, v/v)
Temperature	37 °C	37 °C	37 °C	37 °C	37 °C	RT^b^
Light	--^c^	--	--	--	dark	100 ~ 120 umol-photon · m^−2^ · s^−1^
Fermentation time	48 h	84 h	48 h	84 h	84 h	8 d
OD_680_	20.03 ± 1.42	24.83 ± 1.45	20.26 ± 0.15	26.10 ± 0.61	22.91 ± 1.79	2.63 ± 0.09
Fluorescence intensity^d^	106.36 ± 5.32	340.42 ± 19.13	17.46 ± 2.33	115.31 ± 7.60	324.723 ± 32.09	72.93 ± 2.31
Shaking speed	220 rpm	220 rpm	220 rpm	220 rpm	220 rpm	0 rpm
SRA accession number	SRX352462	SRX354137	SRX354139	SRX354141	SRX354143	SRX354142

^a^the transcriptome datasets of these samples were sequenced before

^b^room temperature (25 ± 2 °C)

^c^do not take the influence of light into account

^d^the fluorescence intensity of lipid dyed with nile red

When *C. sorokiniana* was cultivated in light, the fluorescence intensity increased steadily from 12.02 ± 1.85 at third day to 72.93 ± 2.31 at eighth day, increasing by 6.4 times and the OD_680_ increased continuously from 0.11 ± 0.01 to 2.63 ± 0.14 over the whole cultivation period (Fig. 1c). These indicated that *C. sorokiniana* could absorb CO_2_ as carbon source to reproduce and also transform it into lipid accumulated in cells, which provided a promising strategy to alleviate global warming and energy crisis. When cultured heterotrophically in darkness with nitrogen-limited condition, *C. sorokiniana* accumulated lipid as well and the fluorescence intensity increased by 28.89 times at 84 h (324.72 ± 32.09, Fig. 1d). Compared with photoautotrophy, heterotrophy could make *C. sorokiniana* yield more biomass and achieve higher lipid productivity (Fig. 1c, d).

### Sequencing and *de novo* assembly

**Fig. 2:**
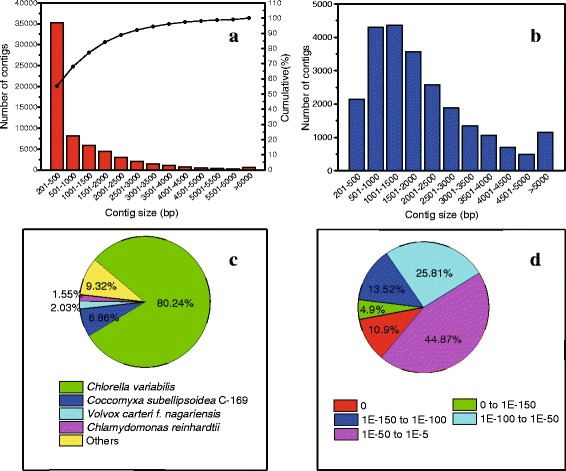
Characteristics of homology search of assembly against the Nr database. **a**: The length distribution of contigs assembled. **b**: The length distribution of contigs with match (with a cut-off E-value of 1.0E-5) in Nr database. **c**: Species distribution of the total homologous sequences with E-value ≤ 1.0E-5. **d**: E-value distribution of Blastx hits for each contigs with E-value ≤ 1.0E-5. We used the first hit of each search results for analysis

After Illumina Hiseq2000 paired-end sequencing, over 244,291,069 raw reads were generated and are available at the NCBI SRA database (Table 1). All the raw reads were subjected to trimming based on base quality score and read length, and 229,288,757 clean reads were generated (Additional files 1 and 2), which were *de novo* assembled into 72,902 contigs with N50 of 2,502 bp. After clustered, 63,811 non-redundant contigs, ranging from 200 bp to 15,932 bp, were generated with an average length of 1,022 bp (Fig. 2a, Additional file 3), which was used for the following analysis. The Transcriptome Short Assembly project has been deposited at DDBJ/EMBL/GenBank under the accession GAPD00000000. The version described in this paper is the first version GAPD01000000.

### Annotation of contigs

**Fig. 3:**
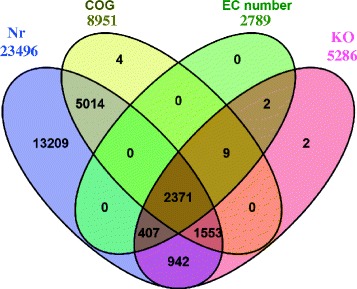
Venn diagram of annotated contigs’ number in different database. Nr: NCBI’s non-redundant database. COG: Clusters of Orthologous Groups (COG) database. EC number and KO: the annotation results of KEGG database. Each figure means the number of annotated contigs in corresponding databases

After compared against the NCBI’s Nr database using Blastx, 23,496 contigs (36.8 % of total contigs) were found having homologous sequence in Nr database (Fig. 3, Additional file 4). Due to the lack of genome information, a large proportion of the contigs (40298, 63.2 %) could not be matched to homologous sequence in any database, among which 10,471 potential coding regions were predicted using Transdecoder (Additional file 5). These predicted coding regions seem to be new genes, and their functions should be further confirmed. EC number and KO identifier were also assigned from the annotation results of KEGG, and 2,789 contigs were assigned with EC number (Fig. 3, Additional file 4). There were 2,371 contigs which were all matched with homologous sequences in all the databases used (Fig. 3). Particularly, the length of most contigs with homologous sequence in Nr database were between 500 and 2500 bp (14801, 62.79 %) and the match efficiency decreased as the length of contigs increased (Fig. 2b), indicating that most genes of *C. sorokiniana* were in the range of 500 bp and 2500 bp. Moreover, the homologous sequences matched in Nr came from closely related green microalgae species, including *C. variabilis* (80.24 % of all annotated contigs), *Coccomyxa subellipsoidea* C-169 (6.86 %) and *Volvox carteri f. nagariensis* (2.03 %) (Fig. 2c), based on which we selected *Chlorella sp.* NC64A as the candidate for predicting transcription factors. Similar results were also reported in the transcriptomic analysis of *Dunaliella tertiolecta* [16] and *Chlamydomonas moewusii* [20]. The E-value distribution of the top match in Nr database showed that 55.13 % of the matched sequences had E-value ≤ 1.0E-50, and 44.87 % ranged from 1.0E-5 to 1.0E-50 (Fig. 2d). Similar results were also reported in the *de novo* transcriptomic analysis of *Ambystoma mexicanum* [21].

### Function classification and Transcription factor analysis

**Fig. 4:**
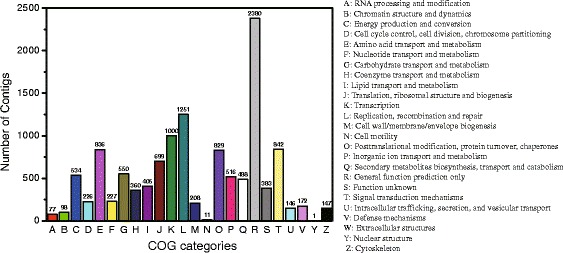
Histogram presentation of clusters of orthologous groups (COG) categories. Out of 23496 Nr hits, 8951 contigs were assigned at least one COG category among the 24 categories

8,951 contigs were assigned with at least 1 COG category and 13 contigs had no homologous sequence in Nr database but matched homologous sequence in COG database (Fig. 3). Among the 24 COG categories, the cluster for “general function prediction only” accounted for the largest group (2380, 19.22 %), followed by “Replication, recombination and repair” (1251, 10.1 %) and “Transcription” (1000, 8.07 %). In addition, the following categories contained the minimum number of contigs: “Nuclear structure” (1), “Cell motility” (11, 0.0009 %), “RNA processing and modification” (77, 0.006 %). We even did not find any contig assigned to the category “Extracellular structures” (Fig. 4, Additional file 4).

Transcription factors, most of which were protein, also play significant roles in responding to environment stress by regulating gene expression which were classified into different families such as SBP, C3H and bHLH. After searching against the transcription factors database of *Chlorella sp.* NC64A [22], 203 contigs were found having homologous sequences and assigned into 12 families (Table 2, Additional file 4). The most abundant transcription factor family was SBP family related to flower development in plant [23]. C3H family was the next abundant transcription factor family, which played an important role during *Arabidopsis* embryogenesis and functioned primarily in the apical domain of the embryo [24] (Table 2). In terms of the specific function of these transcription factor families in *C. sorokiniana*, further studies should be further conducted. Moreover, the differential expression of transcription factor were also investigated. 27 contigs assigned to 20 different transcription factors were found at least 2-fold up-regulated while only 4 contigs homologous with 4 different transcription factors at least 2-fold down-regulated in nitrogen-limited condition. When *C. sorokiniana* was cultivated in light, we found 23 contigs homologous with 12 transcription factors at least 2-fold up-regulated and 40 contigs annotated as 17 transcription factors at least 2-fold down-regulated (Table 3, Additional file 4).

**Table 2:** Transcription factor families identified in *C. sorokiniana*

Transcription factor family	Number of contigs	Number of TF ID^a^
ARR-B	1	1
bHLH	3	2
C2H2	6	2
C3H	21	13
CPP	6	2
E2F-DP	4	2
G2-like	11	6
HB	11	2
MYB	4	3
MYB-related	139	6
SBP	84	20
WRKY	2	1

^a^Transcription factor ID. In the Transcription Factor Database (PlnTFDB), each transcriptome factor family contains several items and each item assigned with a TF ID

**Table 3:** The transcription factors with at least 2-fold expression change

Transcription factor ID	Transcription factor family	Transcription factor ID	Transcription factor family
up-regulated* in nitrogen-limited condition		up-regulated in light	
IGS.gm_27_00071	bHLH	fgenesh3_pg.C_scaffold_21000059	C2H2
gw1.16.195.1	C3H	IGS.gm_27_00067	C3H
IGS.gm_27_00067	C3H	estExt_fgenesh3_pg.C_130033	C3H
IGS.gm_5_00352	C3H	IGS.gm_32_00066	C3H
IGS.gm_14_00202	C3H	IGS.gm_15_00154	G2-like
IGS.gm_21_00108	E2F-DP	IGS.gm_5_00099	G2-like
IGS.gm_16_00059	G2-like	IGS.gm_5_00463	G2-like
IGS.gm_5_00099	G2-like	estExt_fgenesh3_pg.C_240053	MYB_related
IGS.gm_15_00154	G2-like	IGS.gm_28_00074	SBP
estExt_fgenesh3_pg.C_10352	HB	IGS.gm_22_00154	SBP
fgenesh3_pg.C_scaffold_28000005	MYB_related	IGS.gm_21_00140	SBP
gw1.9.308.1	MYB_related	IGS.gm_27_00028	SBP
estExt_fgenesh3_pg.C_10164	MYB_related		
IGS.gm_28_00074	SBP	down-regulated in light	
fgenesh3_pg.C_scaffold_6000074	SBP	IGS.gm_5_00352	C3H
IGS.gm_17_00084	SBP	IGS.gm_3_00649	C3H
IGS.gm_21_00140	SBP	gw1.8.374.1	C3H
IGS.gm_17_00054	SBP	IGS.gm_26_00138	CPP
estExt_fgenesh3_pg.C_130036	SBP	estExt_fgenesh3_pg.C_10352	HB
fgenesh3_pg.C_scaffold_2000108	SBP	IGS.gm_4_00164	HB
		gw1.9.308.1	MYB_related
down-regulated^#^ in nitrogen-limited condition		estExt_Genewise1Plus.C_380072	SBP
estExt_fgenesh3_pg.C_130033	C3H	fgenesh3_pg.C_scaffold_9000137	SBP
estExt_Genewise1Plus.C_380072	SBP	gw1.9.427.1	SBP
estExt_fgenesh3_pg.C_300018	SBP	IGS.gm_13_00158	SBP
estExt_fgenesh3_pg.C_130070	SBP	IGS.gm_17_00054	SBP
		IGS.gm_17_00084	SBP
		fgenesh3_pg.C_scaffold_25000062	SBP
		estExt_fgenesh3_pg.C_130070	SBP
		fgenesh3_pg.C_scaffold_6000074	SBP
		estExt_fgenesh3_pg.C_130036	SBP

*means at least 2-fold up-regulation

^#^means at least 2-fold down-regulation

Up to now, it have been proved that Dof-type transcription factor and bHLH family have the function of regulating lipid accumulation in plants [25–27]. In this study, two transcription factors (IGS.gm_27_00071 and IGS.gm_8_00085) in bHLH family were identified and found both up-regulated in nitrogen-limited condition, which further confirm the significance of bHLH family in the in lipid accumulation (Additional file 4). At the same time, others transcription factors assigned to other families were also found with at least 2-fold expression change in respond to nitrogen deprivation (Table 3), and most of these transcription factor families were also reported to be up-regulated in *Chlamydomonas reinhardtii* cultivated in N-deprived condition especially the MYB-related, SBP and C3H family [28, 29]. Thus, regulating these transcription factors would be a potential approach to increase the lipid accumulation [30, 31]. Moreover, many transcription factors related to photosynthetic carbon fixation were also found to be up-regulated or down-regulated in light (Table 3, Additional file 4). These results would be very useful for the improvement of photosynthetic carbon assimilation in microalgae as few transcription factors involving in photosynthetic carbon assimilation were investigated [32–34].

### Genes expression quantification

**Fig. 5:**
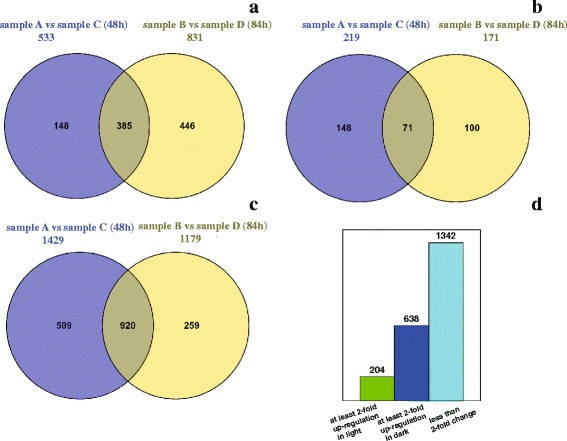
Differential genes expression in *C. sorokiniana* of six cultivation conditions. **a**: At least 2-fold up-regulation in nitrogen-limited condition. **b**: At least 2-fold down-regulation in nitrogen-limited condition. **c**: No difference between nitrogen-limited and nitrogen-replete condition (<2-fold). **d**: Differential genes expression between light and dark condition

The expression abundance of genes obtained from the annotation of assembled contigs were quantified using FPKM method [35]. When comparing genes expression abundance of sample A (nitrogen-limited condition, 48 h) with that of sample C (nitrogen-replete condition, 48 h), we found 533 genes were at least 2-fold up-regulated and 219 genes were at least 2-fold down-regulated in nitrogen-limited condition (Fig. 5, Additional file 6). Then comparing genes expression abundance between sample B (nitrogen-limited condition, 84 h) and sample D (nitrogen-limited condition, 84 h), 831 genes were found at least 2-fold up-regulated, while 171 genes down-regulated in sample B (Fig. 5, Additional file 6).

We also investigated the gene expression profiles for cultivation with 48 h and 84 h, and found 385 genes at least 2-fold up-regulated in nitrogen-limited condition at both cultivation times (Fig. 5a), while 71 genes at least 2-fold down-regulated (Fig. 5b). The expression change of most genes (1429 genes at 48 h, 1179 genes at 84 h, respectively) were less than 2-fold, and 920 genes were found identical at both time (Fig. 5c). Interestingly, we found more genes with at least 2-fold up-regulation and less genes with at least 2-fold down-regulation at 84 h compared with the counterparts at 48 h (Fig. 5a b). The reason for this may be the concentration of nitrogen in the media declined with *C. sorokiniana* growing, which could induce more genes to increase its transcriptional level as a response to the lower concentration of nitrogen.

To investigate the differential expression of genes related to lipid accumulation, we mainly focused on the lipid-related metabolic pathways, including fatty acid biosynthesis and catabolism pathway, triacylglycerol biosynthesis pathway, and starch biosynthesis and catabolism pathway (Table 4). In these metabolic pathways, we found most genes up-regulated in nitrogen-limited condition at both 48 h and 84 h except those in starch metabolic pathway. On the contrary, the starch biosynthesis pathway was down-regulated in nitrogen-limited condition, while the starch catabolic pathway was up-regulated, which was also reported in other microalgae [15], indicating microalgae preferred to synthesis lipid rather than starch in nitrogen-limited condition.

**Table 4:** Differential gene expression in lipid accumulation related pathways

Product	Gene name	EC number	Trinity ID	Log_2_FC^a^ (48 h)	Log_2_FC (84 h)
Fatty acid biosynthyesis pathway					
acetyl-CoA carboxylase	ACC	EC:6.4.1.2	comp6756_c0_seq1	−1.71	−0.63
biotin carboxylase	BC	EC:6.3.4.14	comp12323_c26_seq1	0.82	2.55
malonyl-CoA ACP transacylase	MAT	EC:2.3.1.39	comp11367_c0_seq2	−1.27	−0.74
KAS Beta-ketoacyl-ACP synthase	KASII	EC:2.3.1.179	comp11672_c1_seq3	0.48	0.72
KAS Beta-ketoacyl-ACP synthase	KASIII	EC:2.3.1.180	comp10141_c0_seq2	0.27	0.35
Beta-ketoacyl-ACP reductase	KAR	EC:1.1.1.100	comp10720_c0_seq1	1.23	0.74
beta-hydroxyacyl-ACP dehydrase	HAD	EC:4.2.1.59	comp12130_c0_seq1	0.31	1.43
Enoyl-ACP reductase	EAR	EC:1.3.1.10	comp5575_c1_seq1	−2.23	0.10
acyl-ACP desaturase	AAD	EC:1.14.19.2	comp12618_c0_seq1	0.27	0.45
Oleoyl-ACP thioesterase	OAT	EC:3.1.2.14	comp10287_c1_seq1	0.54	0.53
Triacylglycerol biosynthesis pathway					
glycerol kinase	GK	EC:2.7.1.30	comp17390_c0_seq1	1.21	1.12
glycerol-3-phosphate O-acyltransferase	GPAT	EC:2.3.1.15	comp8649_c0_seq4	0.14	2.41
1-acyl-sn-glycerol-3-phosphate acyltransferase	AGPAT	EC:2.3.1.51	comp8576_c1_seq1	1.05	0.99
phosphatidate phosphatase	PP	EC:3.1.3.4	comp10073_c0_seq1	−0.75	−3.36
diacylglycerol O-acyltransferase	DGAT	EC:2.3.1.20	comp8627_c0_seq2	0.08	0.15
Fatty acid catabolism pathway					
acyl-CoA synthetase	ACSL	EC:6.2.1.3	comp11861_c1_seq8	−0.57	−0.29
acyl-CoA oxidase	AOx	EC:1.3.3.6	comp8247_c0_seq2	2.17	1.24
acyl-CoA dehydrogenase	ACDH	EC:1.3.8.7	comp12261_c15_seq6	0.62	1.22
enoyl-CoA hydratase	ECH	EC:4.2.1.17	comp8664_c0_seq1	0.46	0.27
3-hydroxyacyl-CoA dehydrogenase	HADH	EC:1.1.1.35, 1.1.1.211	comp13763_c0_seq1	1.64	1.97
acetyl-CoA C-acyltransferase	ACAT	EC:2.3.1.16, 2.3.1.9	comp12241_c5_seq15	0.89	0.02
Starch biosynthesis and catabolism, and ethanol fermentation pathway					
phosphoglucomutase	PGM	EC:5.4.2.2	comp10999_c2_seq3	−1.11	−0.12
ADP-glucose pyrophosphorylase	AGPase	EC:2.7.7.27	comp11635_c1_seq2	0.30	1.67
starch synthase	SS	EC:2.4.1.21	comp13246_c0_seq1	0.04	0.30
1,4-α-glucan branching enzyme	BE	EC:2.4.1.18	comp7752_c1_seq1	−1.47	−1.66
hexokinase	HXK	EC:2.7.1.1	comp5345_c0_seq1	−0.20	1.49
β-amylase	β-AMY	EC:3.2.1.2	comp13107_c0_seq1	1.49	1.36
α-amylase	α-AMY	EC:3.2.1.1	comp11995_c1_seq7	2.22	1.10
oligo-1,6-glucosidase	O1,6G	EC:3.2.1.10	comp11995_c1_seq11	2.20	1.49
starch phosphorylase	Spase	EC:2.4.1.1	comp12085_c7_seq13	0.79	1.47
pyruvate decarboxylase	PDC	EC:4.1.1.1	comp6820_c0_seq2	−0.30	0.93
alcohol dehydrogenase	ADH	EC:1.1.1.1	comp11611_c1_seq3	0.71	1.13
Pyruvate dehydrogenase complex	PDHC	EC:1.2.4.1, 2.3.1.12, 1.8.1.4	comp12893_c0_seq1	1.48	1.90

^a^ $$ \mathrm{Log}2\mathrm{F}\mathrm{C}= Log2\left(\frac{FPKMnitrogen- limited}{FPKMnitrogen- replete}\right) $$

When investigating differential gene expression between light and dark condition, we found 842 genes with expression change more than 2-fold, comprising 204 genes up-regulated in light and 638 genes up-regulated in dark. Similarly, the expression change of most genes (1342 genes) were less than 2-fold (Fig. 5d). In terms of photosynthetic carbon fixation, we particularly focused on the genes involving in Calvin cycle and found all genes except TPI (coding triosephosphate isomerase) up-regulated in light. The PGK (coding phosphoglycerate kinase), RBCL (coding ribulose-bisphosphate carboxylase large chain) and RPK (coding phosphoribulokinase) even found up-regulated by 10.6, 5.42 and 4.66 times, respectively (Table 5). Unfortunately, there were still many genes with differential expression annotated as “hypothetical protein with unknown functions” in the annotation results (Additional file 6). Therefore, it will be necessary to investigate the potential functions of these genes.

**Table 5:** Differential gene expression in Calvin cycle

Product	Gene name	EC number	Trinity ID	Log_2_FC^a^
ribulose-bisphosphate carboxylase large chain	RBCL	EC:4.1.1.39	comp10529_c0_seq1	2.44
fructose-bisphosphate aldolase, class I	ALDO	EC:4.1.2.13	comp12064_c4_seq2	1.41
sedoheptulose-1,7-bisphosphatase	SEBP	EC:3.1.3.37	comp8533_c0_seq1	1.22
transketolase	TRK	EC:2.2.1.1	comp12612_c0_seq1	0.35
ribose 5-phosphate isomerase A	RPIA	EC:5.3.1.6	comp14985_c0_seq1	0.57
phosphoribulokinase	RPK	EC:2.7.1.19	comp13013_c0_seq1	2.22
ribulose-phosphate 3-epimerase	RPE	EC:5.1.3.1	comp12802_c0_seq1	1.65
phosphoglycerate kinase	PGK	EC:2.7.2.3	comp11827_c0_seq2	3.41
glyceraldehyde-3-phosphate dehydrogenase	GAPDH	EC:1.2.1.13	comp11943_c0_seq2	1.75
triosephosphate isomerase	TPI	EC:5.3.1.1	comp8453_c0_seq1	−0.29
fructose-1,6-bisphosphatase	FBPase	EC:3.1.3.11	comp10769_c0_seq3	1.56

^a^ $$ \mathrm{Log}2\mathrm{F}\mathrm{C}= Log2\left(\frac{FPKM\  light}{ FPKM dark}\right) $$

### Real-time quantitative PCR analysis

**Fig. 6:**
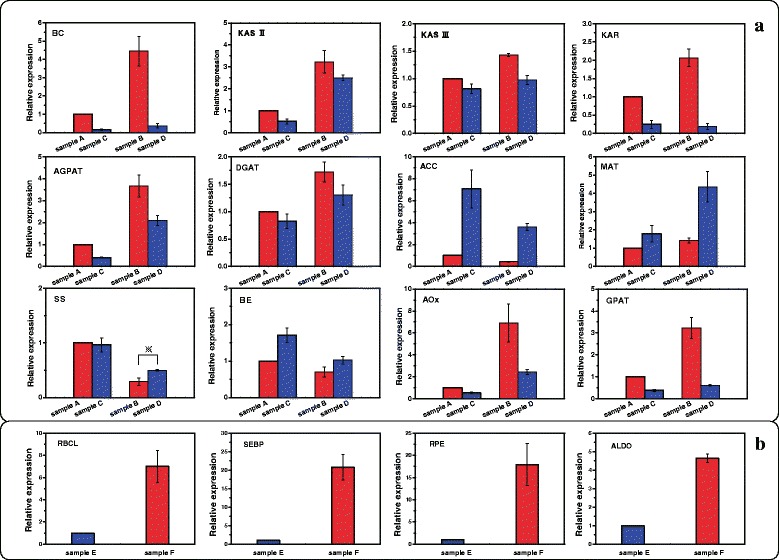
The differentially expressed genes profiles detected by RT-qPCR. *C. sorokiniana* was cultivated under six different conditions. **a**: genes involving in lipid accumulation; **b**: genes involving in Calvin cycle. BC: biotin carboxylase; KAS II: 3-oxoacyl-[acyl-carrier-protein] synthase II; KAS III: 3-oxoacyl-[acyl-carrier-protein] synthase III; KAR: Beta-ketoacyl-ACP reductase; AGPAT: 1-acyl-sn-glycerol-3-phosphate acyltransferase; DGAT: diacylglycerol O-acyltransferase; ACC: acetyl-CoA carboxylase; MAT: malonyltransferase; SS: starch synthase; BE: 1,4-α-glucan branching enzyme; AOx: acyl-CoA oxidase; GPAT: glycerol-3-phosphate O-acyltransferase; RBCL: ribulose-bisphosphate carboxylase large chain; SEBP: sedoheptulose-bisphosphatase; RPE: ribulose-phosphate 3-epimerase; ALDO: fructose-bisphosphate aldolase. Standard error of mean for three technical replicates is represented by the error bars

16 genes were selected to perform Real-time quantitative PCR (RT-qPCR). In the lipid metabolic pathways (Fig. 6a), 6 genes (biotin carboxylase, BC; 3-oxoacyl-[acyl-carrier-protein] synthase II, KAS II; 3-oxoacyl-[acyl-carrier-protein] synthase II, KAS III; Beta-ketoacyl-[acyl-carrier-protein] reductase, KAR; 1-acyl-sn-glycerol-3-phosphate acyltransferase, AGPAT; diacylglycerol O-acyltransferase, DGAT) showed up-regulation in nitrogen-limited condition, especially BC and KAR. However, 2 genes (acetyl-CoA carboxylase, ACC; malonyltransferase, MAT) were found down-regulated in the nitrogen-limited condition. The down-regulation of ACC and up-regulation of BC under nitrogen-limited condition were also reported in *Neochloris oleoabundans* [15].

Moreover, we found the down-regulation of starch biosynthesis pathway (starch synthase, SS; 1,4-α-glucan branching enzyme, BE) in nitrogen-limited condition, indicating that the starch biosynthesis pathway might be inhibited (Fig. 6a). This result was also reported in *N. oleoabundans* [15]. 4 genes involving in carbon fixation pathway (ribulose-bisphosphate carboxylase large chain, RBCL; sedoheptulose-bisphosphatase, SEBP; ribulose-phosphate 3-epimerase, RPE; fructose-bisphosphate aldolase, ALDO) were found all up-regulated in light (Fig. 6b).

Among the 16 genes with RT-qPCR analysis, 15 genes showed the similar expression patterns as those identified by the transcriptomic analysis. Only the gene coding starch synthase showed inconsistent result in term of differential expression between the RT-qPCR analysis and transcriptomic analysis at 84 h. Based on the result of RT-qPCR analysis, this gene was found down-regulated in nitrogen-limited condition at 84 h, while the transcriptomic analysis result showed it was up-regulated in the corresponding condition.

## Conclusions

This study not only provided transcriptome datasets of *C. sorokiniana* under six different conditions, but also new biological insights into the expression of genes associated with lipid accumulation and photosynthetic carbon fixation. Based on our study, it is clear that the application of this approach can contribute to the generation of interesting hypotheses for both fundamental and applied research. Moreover, the *C. sorokiniana*’s transcriptome data could be a contribution for elucidating the physiology and evolution of the chlorophyceans.

## Methods

### Strain and culture conditions for RNA-seq

*C. sorokiniana* (UTEX 1602) was obtained from the Culture Collection of Alga at University of Texas (UTEX, Austin, TX, USA) and cultivated using modified Kuhl medium (Additional file 7). To induce the differential expression of genes involving in lipid accumulation and photosynthetic carbon fixation, six conditions were selected for transcriptome sequencing, including: 0.2 % nitrate supply with cultivation of 48 h (nitrogen-limited, sample A); 0.2 % nitrate supply with cultivation of 84 h (nitrogen-limited, sample B); 0.8 % nitrate supply with cultivation of 48 h (nitrogen-replete, sample C); 0.8 % nitrate supply with cultivation of 84 h (nitrogen-replete, sample D); 0.2 % nitrate supply with cultivation in darkness of 84 h (heterotrophy with nitrogen-limited, sample E); 0.033 % nitrate supply, white fluorescent light (100 ~ 120 μmol photons · m^−2^ · s^−1^) and agitation by air containing 4 % (v/v) CO_2_ with cultivation of 8 d (photoautotrophy with nitrogen-limited, sample F) (Table 1). The transcriptomes of sample A, C, E and F were sequenced in this study and the datasets of sample B and D were sequenced before (SRX354137 and SRX354141, respectively) [19], which we analyze together with the purpose of getting the most comprehensive transcript pool using *de novo* assembly method.

In this study, we selected six experimental conditions to compare the expression level of genes and each experimental condition have one biological replicate (*n* = 1). To keep the concordance of cultivation, the culture method of Sample A, C and E was the same as that of sample B and D [19], using 250 mL Erlenmeyer flask with 100 mL medium shaking at 220 rpm at 37 °C. Sample F was cultivated using Φ1x50 mm Cylindrical glass tube with 300 mL medium agitating with air at room temperature (25 ± 2 °C). After cultivation, cells were harvested by centrifugation (Eppendorf, Germany) at 4000 rpm, for 5 min, at 4 °C. The cell pellets were immediately frozen in liquid nitrogen and stored at −80 °C until further analysis.

### RNA extraction, library construction and sequencing

Total RNA of four samples (sample A, C, E and F) were extracted separately using General Total RNA Extraction Kit (QIAGEN, Germany) according to the manufacturer’s instructions. After the elimination of the contaminant DNA, oligo (dT) beads were used to isolate mRNA from total RNA, followed by mRNA was cut randomly into short fragments. These fragmented RNA was reverse-transcribed to the first-strand cDNA with reverse transcriptase (Invitrogen, USA) that was then used as template to synthesis the second-strand of cDNA with DNA polymeraseIand RNase H (Invitrogen). The resulting short cDNA fragments were purified using QiaQuik PCR Extraction Kit (QIAGEN) and resolved in an elution buffer for end reparation and addition of a single adenine base to 3’ends. Then the cDNA fragments were linked with sequencing adapters and separated in gels by electrophoresis. The fragments with a desirable size were cut from gels and eluted for PCR amplification. After qualified with Agilent 2100, each cDNA library was sequenced with Illumina Hiseq2000 platform (Illumina, USA). These RNA extraction and library construction processes were the same as those used for sample B and D.

### Analysis of biomass and lipid content

The biomass of *C. sorokiniana* was determined by measuring the OD_680_ using the microplate reader (Molecular Devices, USA). Lipid content was determined using the modified nile red staining method [36]. The culture was diluted with corresponding medium until the OD_680_ was between 0.1 and 0.3. Then 1 mL of this algal suspension was stained with 3.33 μL nile red solution (7.8 × 10^−4^ mol · L^−1^ dissolved in acetone) and then excited at 486 nm before measuring the emission at 570 nm using the microplate reader. Glucose concentration was measured using HPLC method (Agilent Technologies, USA).

### Preprocessing, *de novo* assembly and function annotation

The 100 bp paired-end raw reads generated from Illumina Hiseq2000 were analyzed by FastQC tool (v0.10.1) [37] for quality assessment and preprocessed using Python scripts (Additional file 8), including: (a) remove low quality bases with Phred score < 20, (b) remove ambiguous base ‘N’, (c) discard short reads with length < 25 bp. Followed by the high quality reads were *de novo* assembled using Trinity (v2.0.6) [38] with default parameters to construct contigs. Final clustering of contigs were conducted using the Cluster Database at High Identity with Tolerance (CD-HIT) EST suits [39] with minimum similarity cut-off of 90 % to generate the non-redundant contigs used for the following analysis.

For the functional annotation, the non-redundant contigs were searched against with the NCBI's non-redundant (Nr) database and Clusters of Orthologous Groups (COG) database [40, 41] using Blastx algorithm [42] with *E*-value ≤ 10^−5^ and 10^−10^, respectively, and other default parameters. Putative gene function, coding sequence and predicted proteins of corresponding contigs could be obtained by parsing the features of the best hit from each Blastx result. For the contigs that had no hit in any databases, the Transdecoder was used to predict potential coding sequences with default parameters. The Blastx results from COG database were used to identify the cluster of orthologous groups. To identify BRITE functional hierarchies [43], the non-redundant contigs were also submitted to the KEGG Automatic Annotation Server (KAAS) [44] with bi-directional best hit assignment method. KAAS could annotate each submitted sequence with KEGG orthology (KO), corresponding enzyme commission number (EC number) with the threshold of Blast bit scores > 60. Putative transcription factors were also identified by searching Plant Transcription Factor Database (PlnTFDB) [45] using Blastx algorithm [42] with *E*-value Blas^−10^. *Chlorella sp.* NC64A [22] was selected as the candidate to search against in order to predict the transcription factors in *C. sorokiniana*.

### Gene expression quantification

To determine the gene expression abundance, high quality reads from each condition were mapped to the non-redundant contigs to calculate the FPKM value [35] using the RSEM (v1.2.7) [46]. Due to the lack of biological replicates, we selected genes whose FPKM value was greater than 0 in all six conditions to study the differential expression and genes with the change of FPKM value greater than 2-fold in comparison of two different conditions were identified as differential expression.

### Real-time quantitative PCR

In order to avoid the bias caused by the absence of biological replicates, we selected 16 genes, involving in lipid accumulation and carbon fixation, to perform the RT-qPCR. The same conditions were used to cultivate *C. sorokiniana* for the RT-qPCR analysis. M-MLV reverse transcription kit (Promega, USA) was used to synthesis the cDNA according to the manufacturer’s instruction. Gene specific primers (Additional file 9) for RT-qPCR were designed using Vecter NTI software. A 10 μL reaction system was performed on the Eco real-time PCR system (Illumina, USA) with the absolute SYBR Green qPCR Kit Master Mix (Toyobo, Japan) according to the manufacturer’s instruction. The cycle threshold value (CT) was determined and differential expression was calculated using the 2^-△△CT^ method [47] with 18S gene of *C. sorokiniana* as the endogenous reference. Each sample was run in triplicate to confirm the reproducibility of the results.

## Additional files

Additional file 1: General Information about the RNA-Seq Data. (DOCX 20 kb) Additional file 2: Quality assessment of raw and trimmed dataset. (DOCX 7557 kb) Additional file 3: Contigs assembled using Trinity. (RAR 12595 kb) Additional file 4: Annotation of contigs. (XLSX 1984 kb) Additional file 5: Predicted coding sequences. (RAR 1699 kb) Additional file 6: Differential genes expression in *C. sorokiniana*. (XLSX 1025 kb) Additional file 7: Modified Kuhl medium. (DOCX 15 kb) Additional file 8: Python scripts. (DOCX 18 kb) Additional file 9: Primer paired used in RT-qPCR. (XLSX 10 kb)
