# Supplementary material for: De novo transcriptomic analysis of Chlorella sorokiniana reveals differential genes expression in photosynthetic carbon fixation and lipid production
Source: BMC Microbiol. 2016 Sep 26;16:223. doi: 10.1186/s12866-016-0839-8 (PMC5037625; doi:10.1186/s12866-016-0839-8)
Supplement: Additional file 1: — General Information about the RNA-Seq Data. (DOCX 20 kb) [file 12866_2016_839_MOESM1_ESM.docx]

**Additional file 1 General Information of the RNA-seq datasets.**

| **Sample ID** | | **Raw data** | | | **Trimmed data** | | | **High quality leave after trim (%)** |
| --- | --- | --- | --- | --- | --- | --- | --- | --- |
|  |  | **Reads number** | **Reads length (bp)** | **Base number** | **Reads number** | **Reads length (bp)** | **Base number** |  |
| Sample A | _1 | 13,351,261 | 100 | 1,348,477,361 | 11985649 | 25-100 | 1,134,229,145 | 89.8 |
|  | _2 | 13,301,971 | 100 | 1,343,499,071 | 12131630 | 25-100 | 1,005,855,719 | 91.2 |
| Sample B^1^ | _1 | 13,729,355 | 100 | 1,385,654,855 | 13291930 | 25-100 | 1,163,311,877 | 96.8 |
|  | _2 | 13,650,626 | 100 | 1,378,713,226 | 12365400 | 25-100 | 1,022,704,894 | 90.6 |
| Sample C | _1 | 13,574,902 | 100 | 1,366,025,607 | 13158140 | 25-100 | 1,093,503,785 | 96.9 |
|  | _2 | 8,532,092 | 100 | 861,741,292 | 7716903 | 25-100 | 637,364,087 | 90.4 |
| Sample D^1^ | _1 | 10,972,576 | 100 | 1,108,230,176 | 10643081 | 25-100 | 934,803,531 | 97.0 |
|  | _2 | 10,927,006 | 100 | 1,103,627,606 | 9930683 | 25-100 | 823,819,128 | 90.9 |
| Sample E | _1 | 35,814,195 | 100 | 3,617,233,695 | 34707995 | 25-100 | 2,885,910,292 | 97.0 |
|  | _2 | 35,814,195 | 100 | 3,617,233,695 | 33192066 | 25-100 | 2,745,547,007 | 92.7 |
| Sample F | _1 | 37,311,445 | 100 | 3,768,455,945 | 35943453 | 25-100 | 2,945,378,673 | 96.3 |
|  | _2 | 37,311,445 | 100 | 3,768,455,945 | 34221827 | 25-100 | 2,793,299,316 | 91.7 |
| Total |  | 244,291,069 |  | 24,667,348,474 | 229,288,757 |  | 19,185,727,454 | 93.9 |

es

^1^the transcriptome datasets were sequenced before.
