# Supplementary material for: De novo transcriptomic analysis of Chlorella sorokiniana reveals differential genes expression in photosynthetic carbon fixation and lipid production
Source: BMC Microbiol. 2016 Sep 26;16:223. doi: 10.1186/s12866-016-0839-8 (PMC5037625; doi:10.1186/s12866-016-0839-8)

Additional file 2 The quality statistic and length distribution of raw reads and clean reads. For the above chart, the X-axis shows the reads position (bp), and Y-axis shows the quality scores. For the following chart, the X-axis shows the reads length (bp), and Y-axis shows the reads number. Each sample comprise two reads dataset corresponding to the paired-end sequencing.


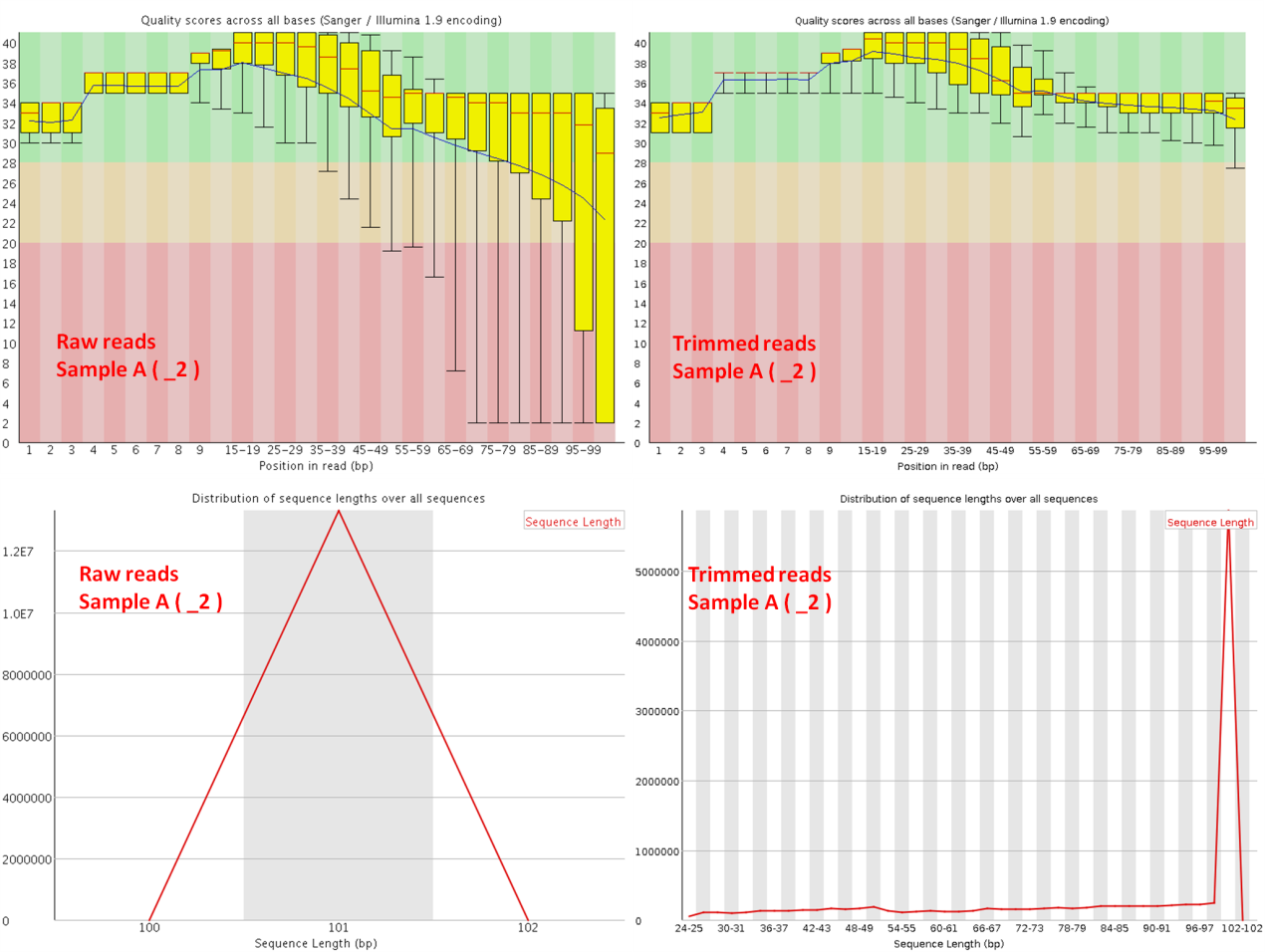

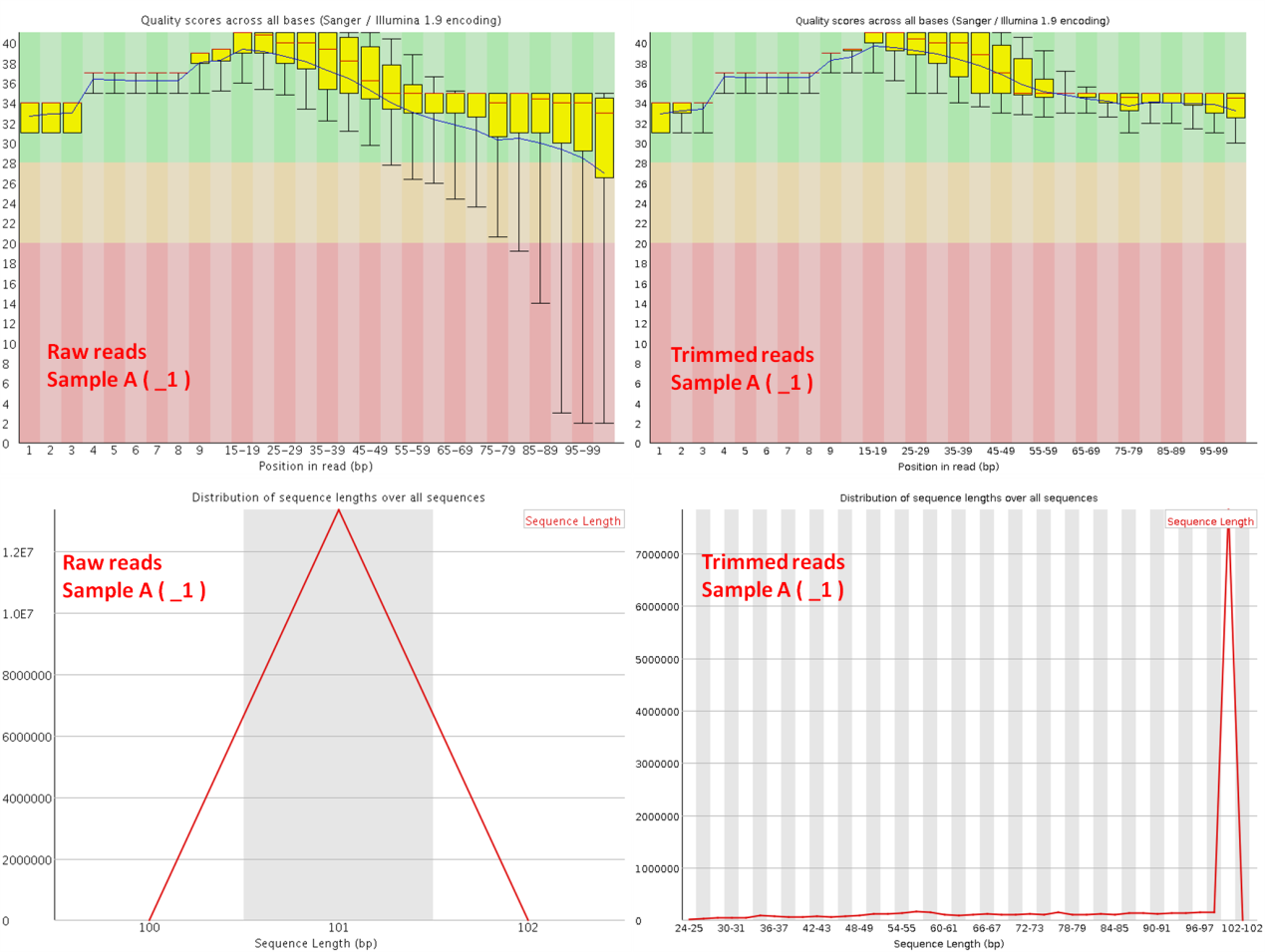


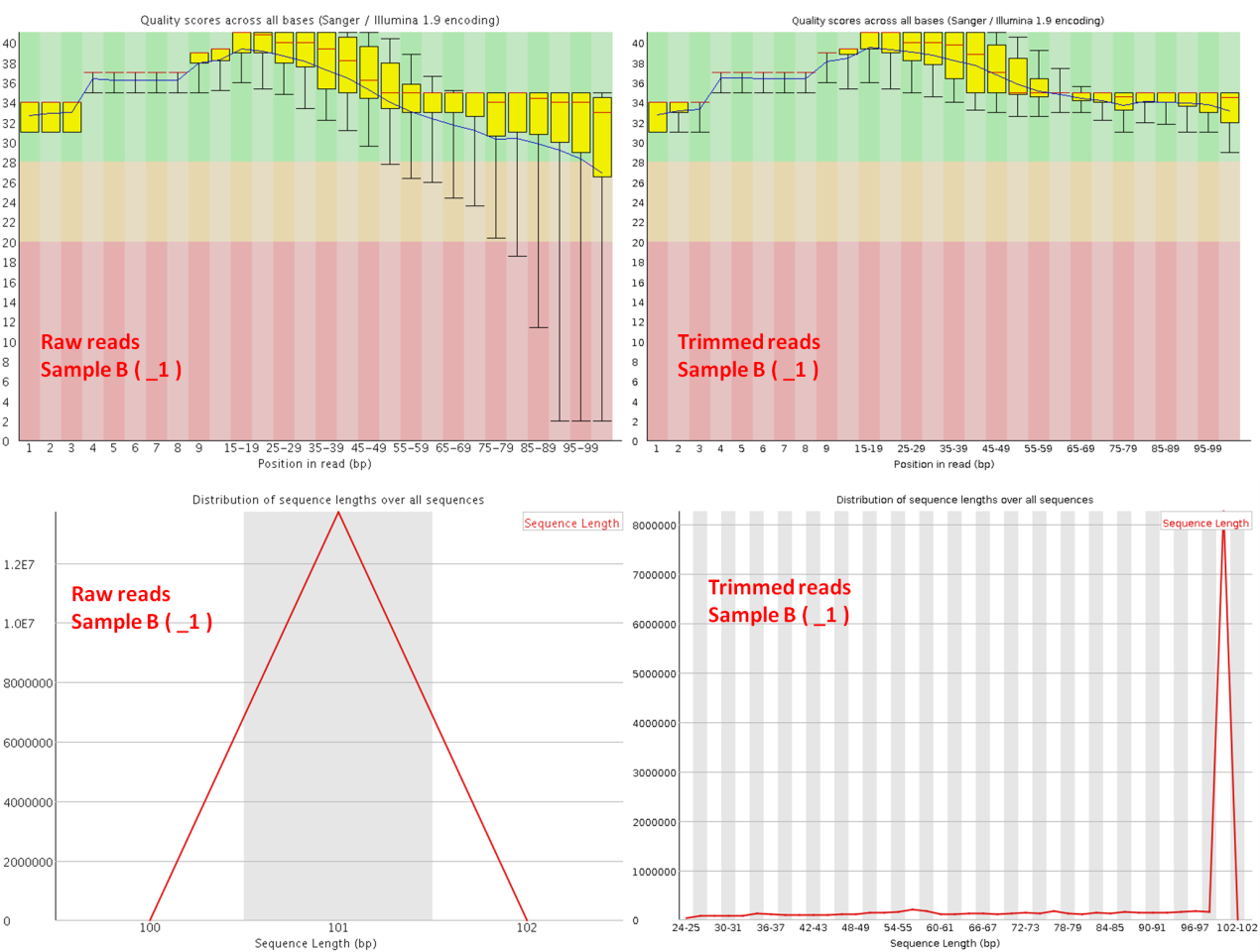

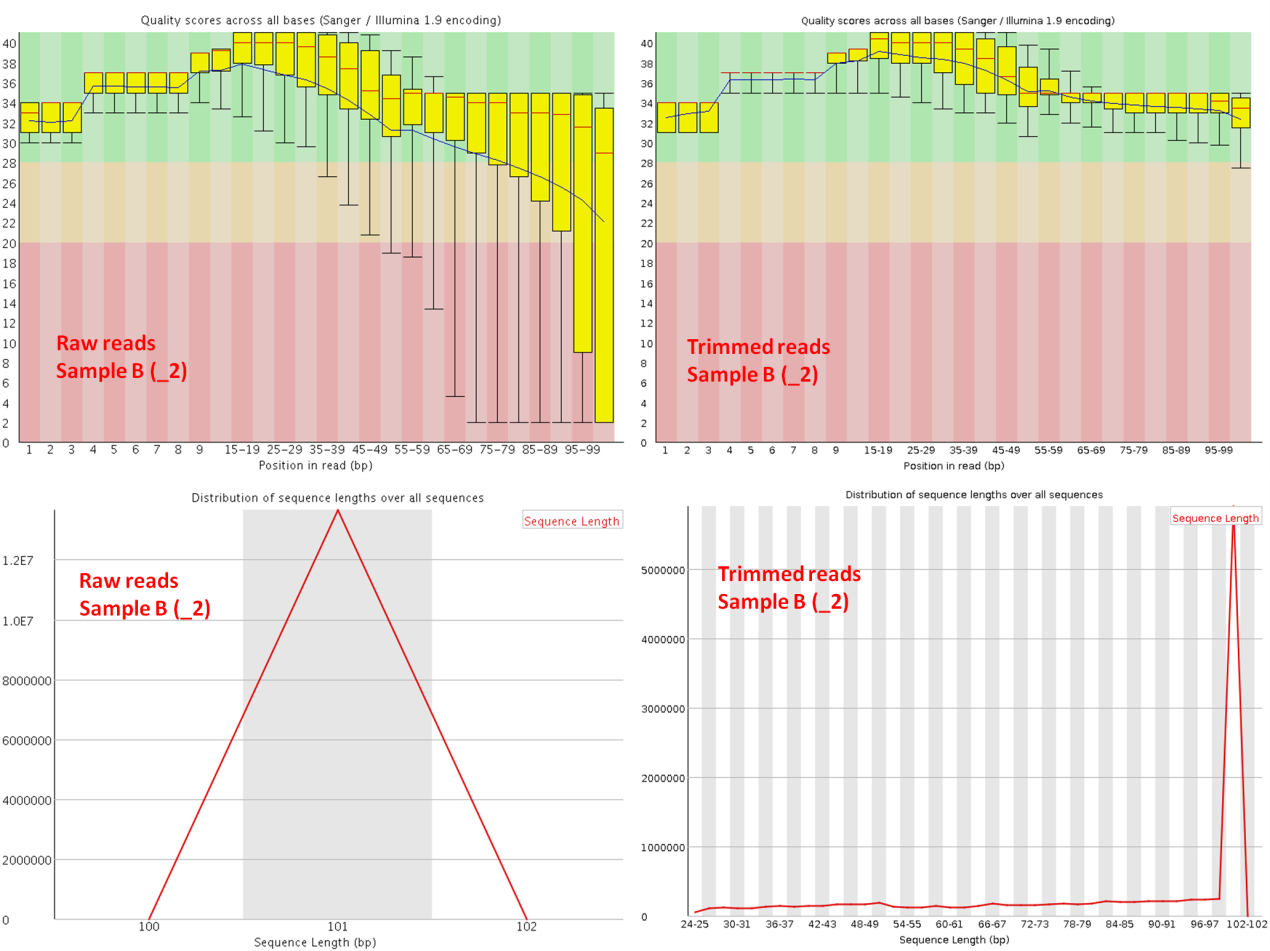


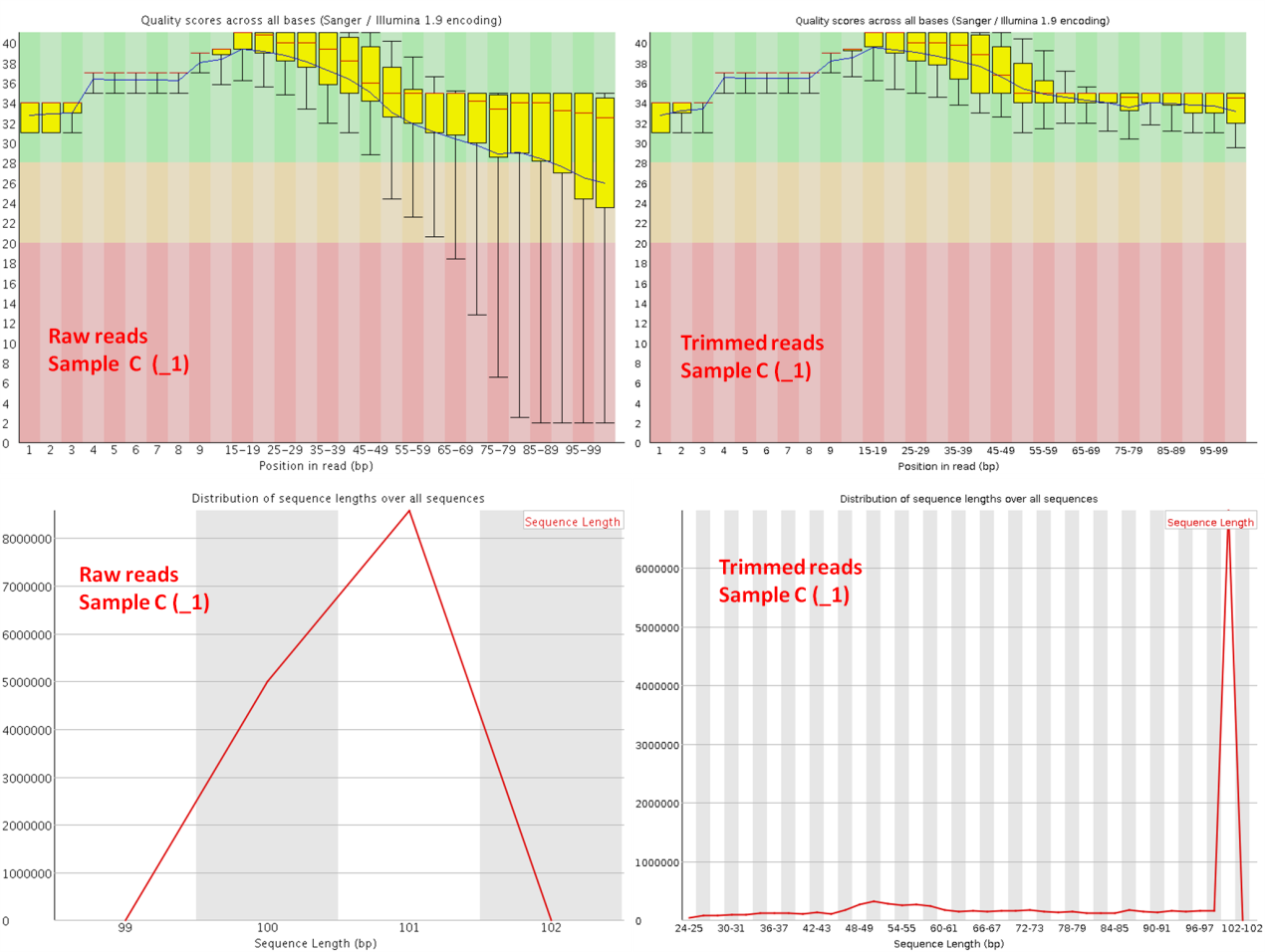

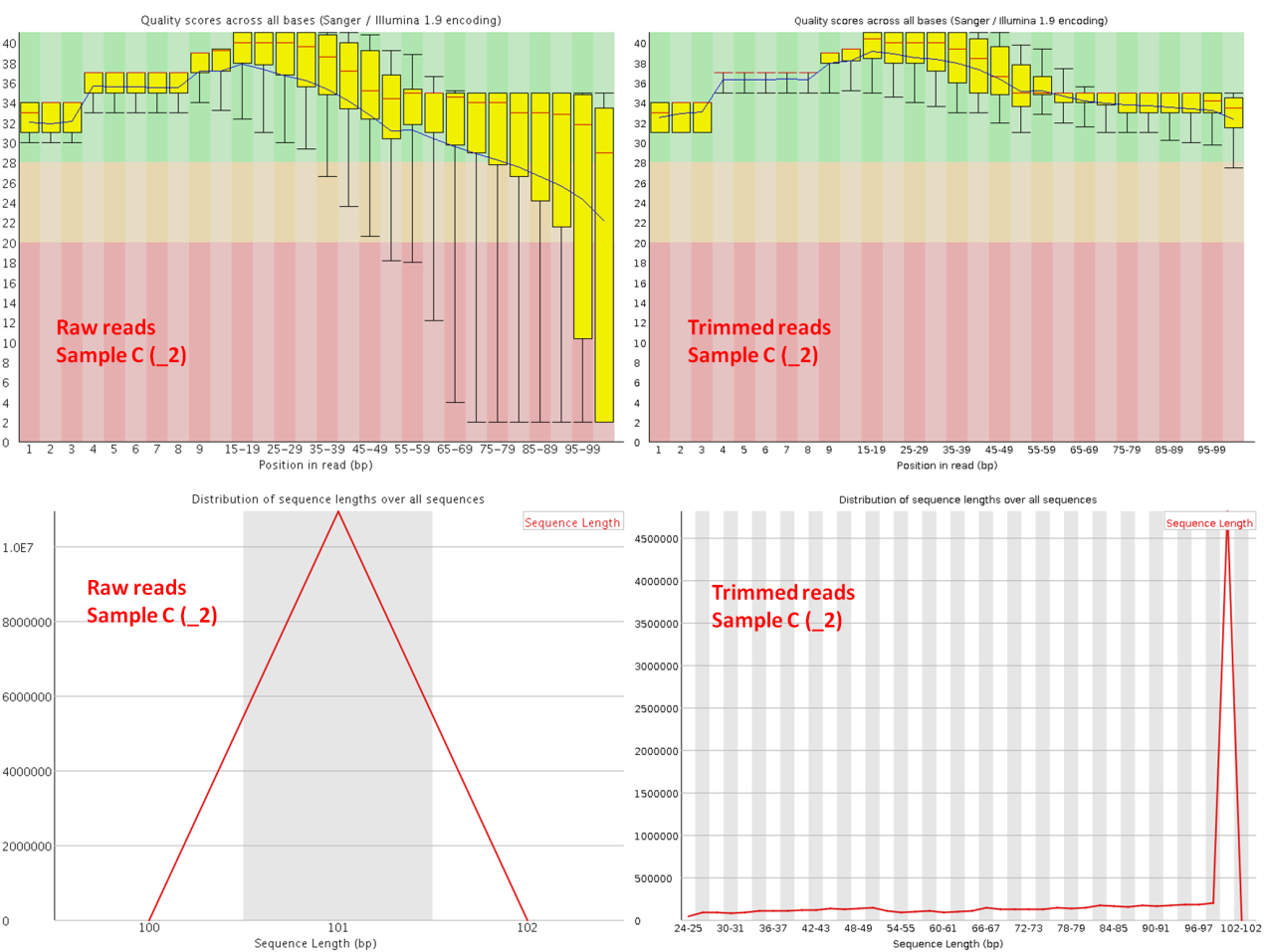


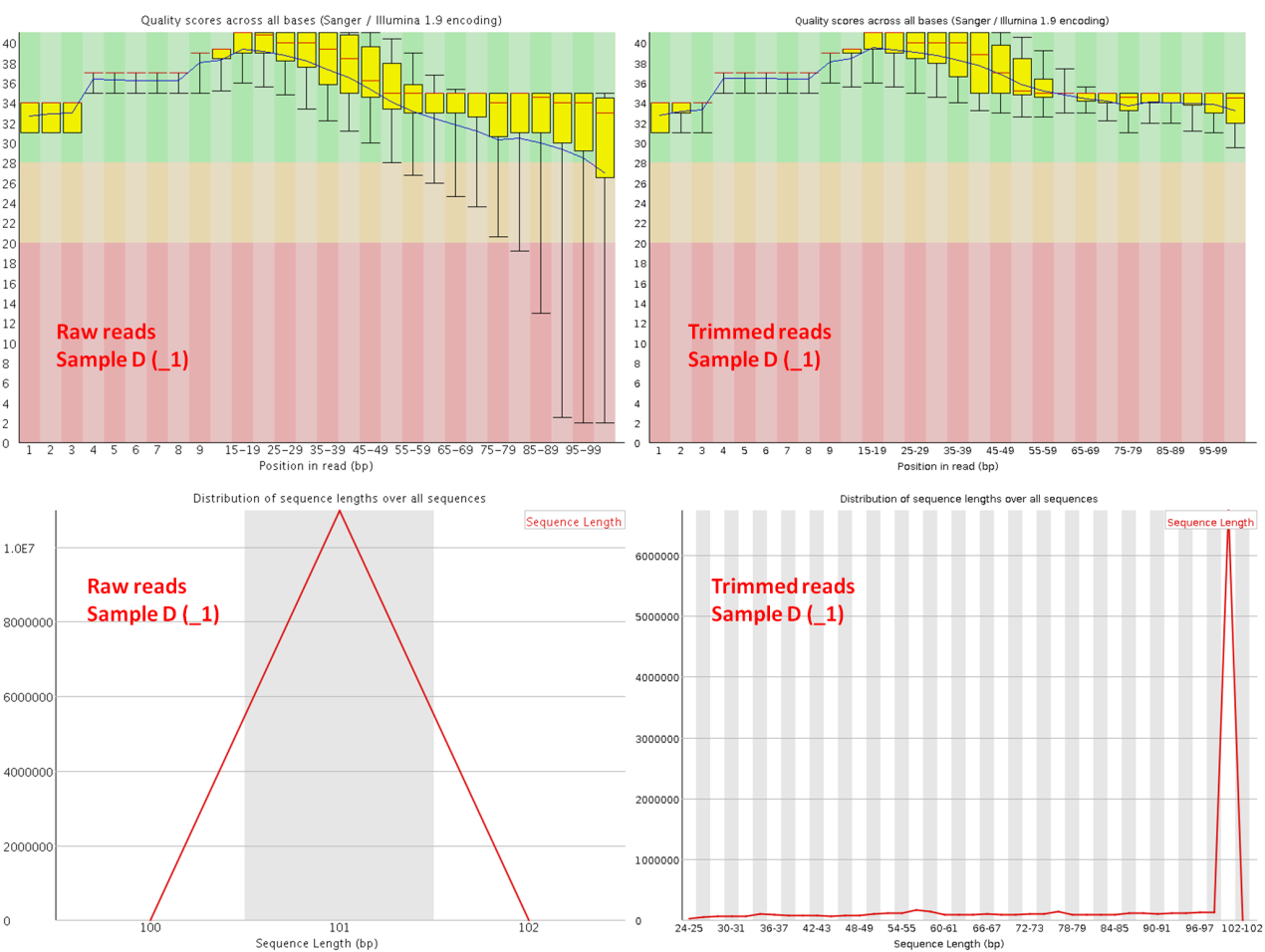

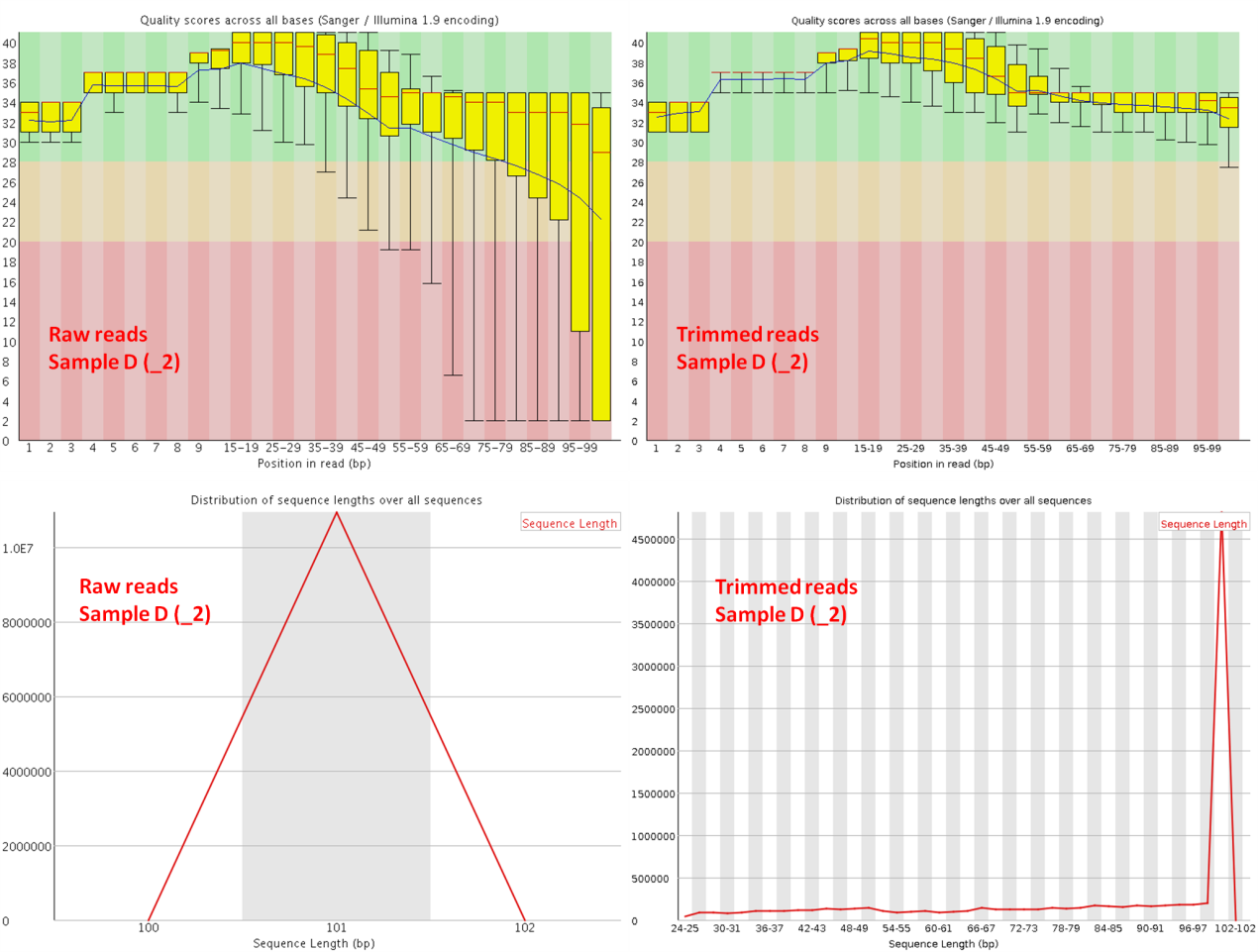


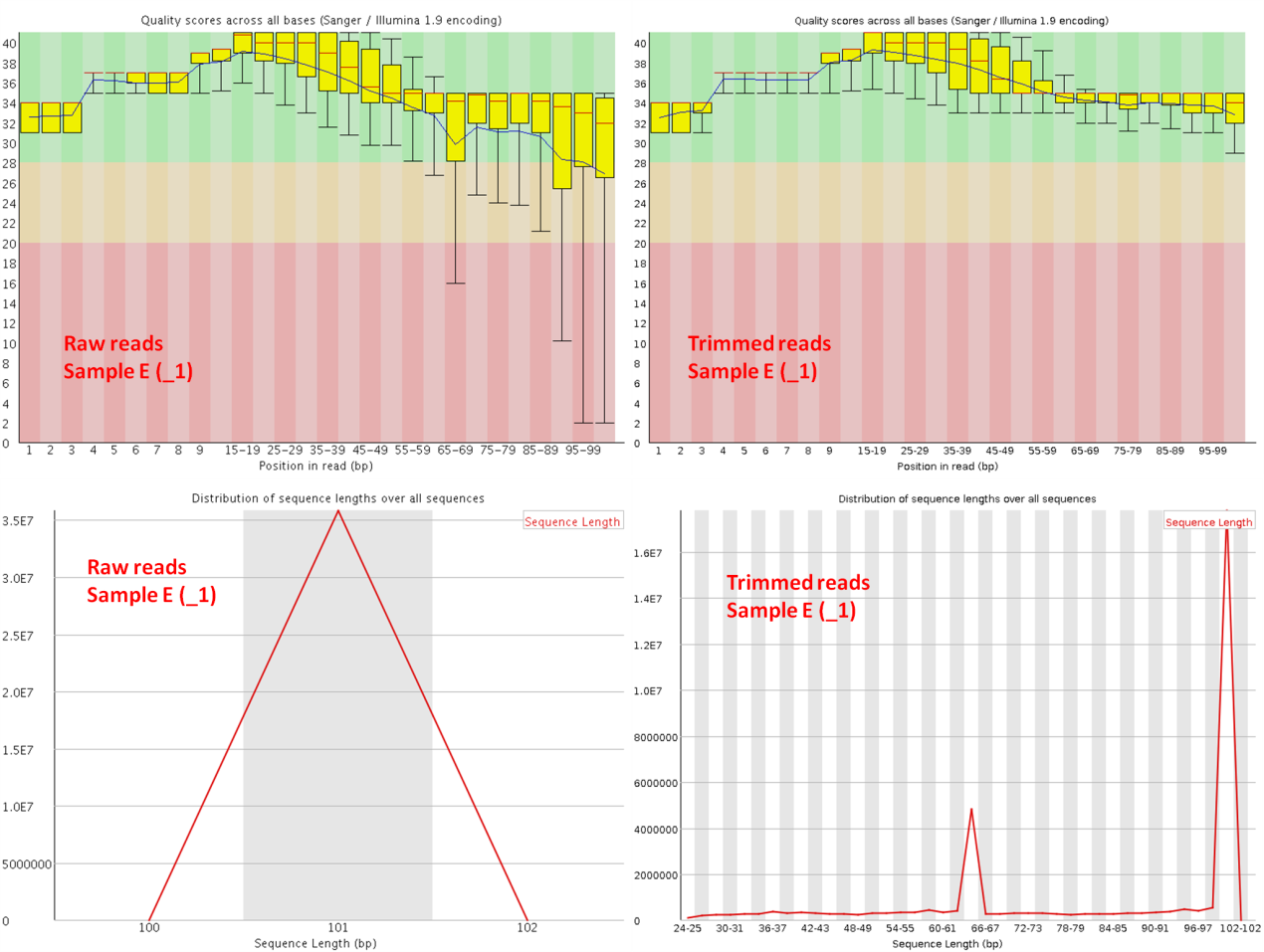

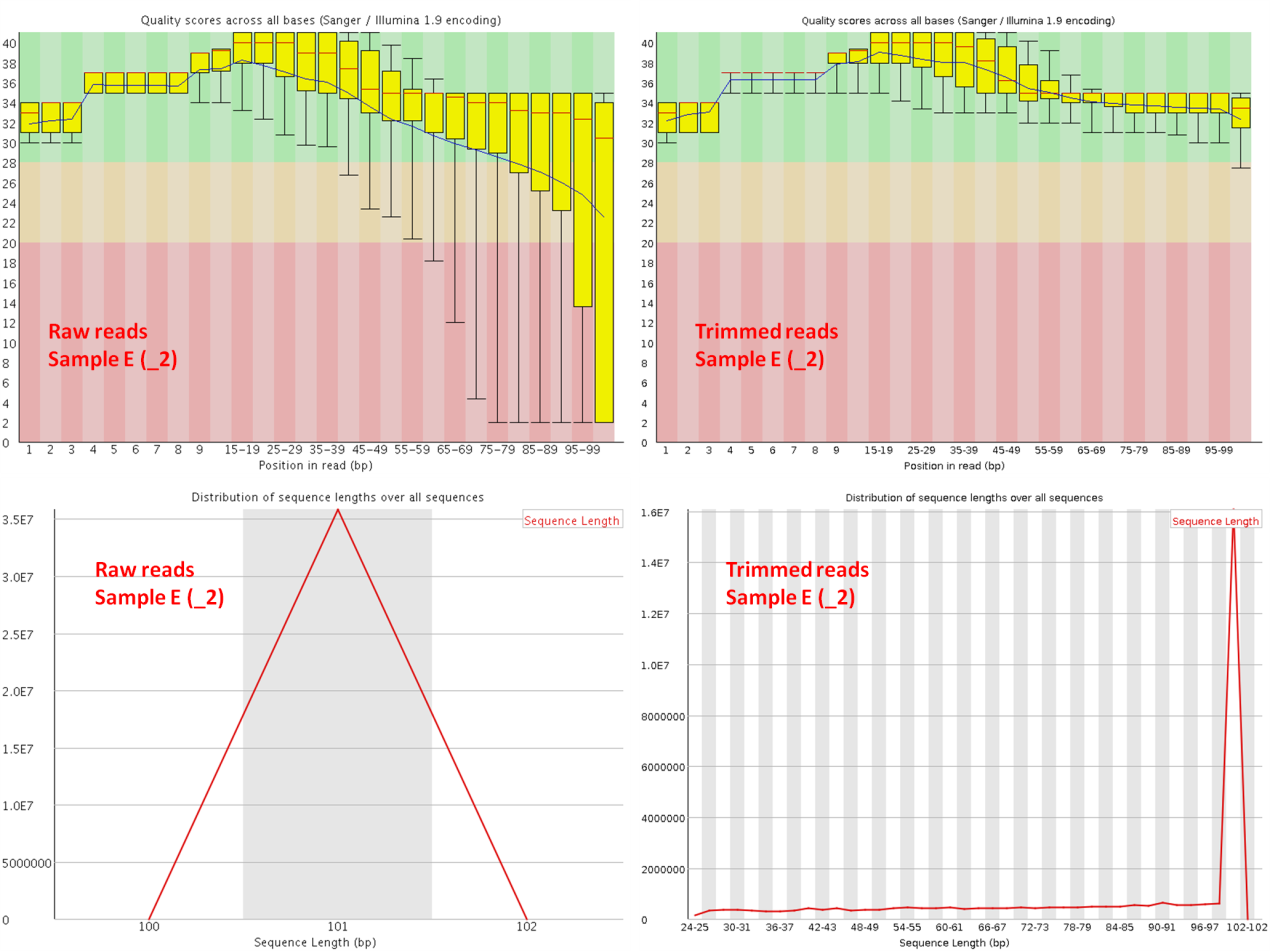


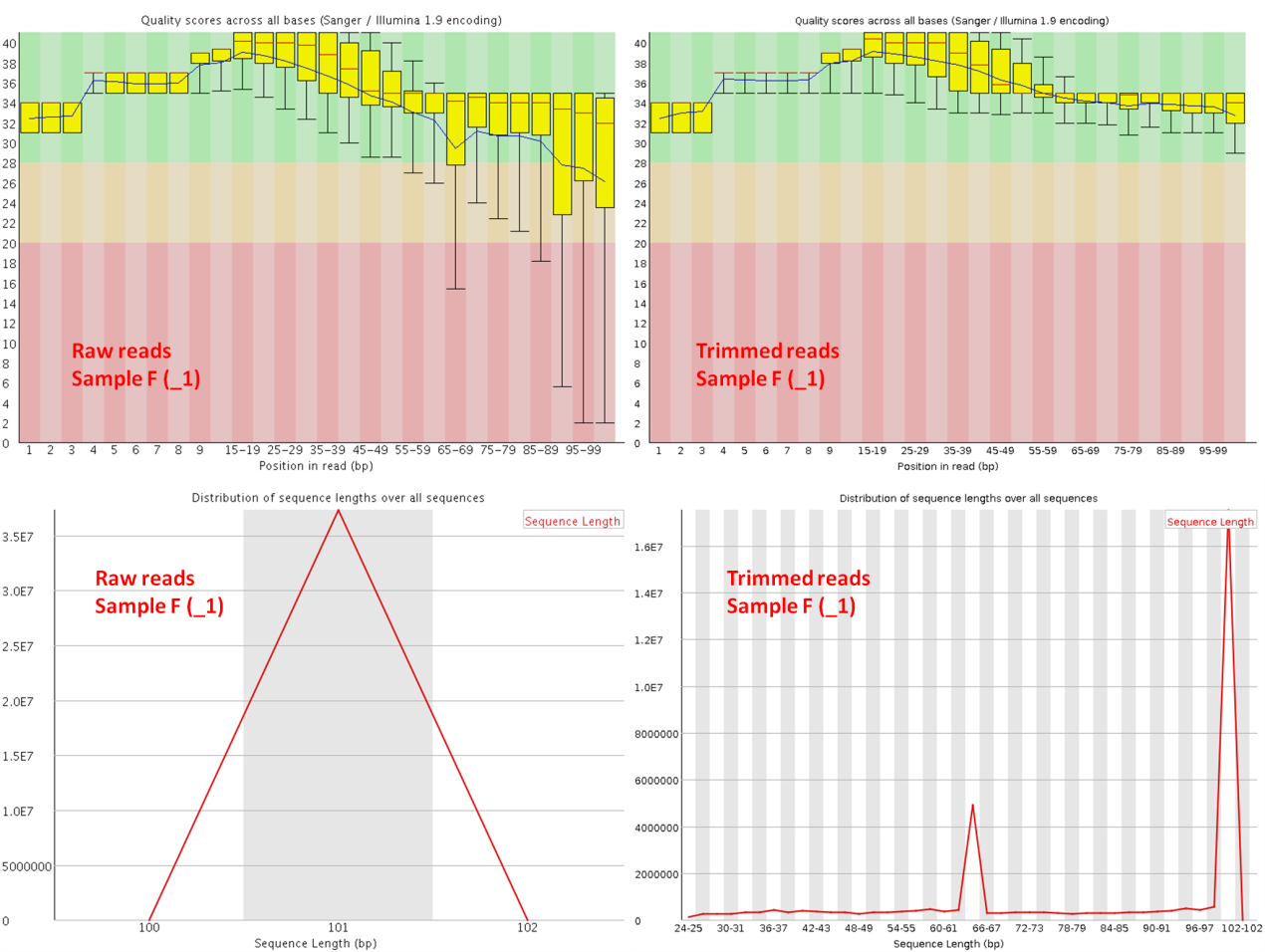

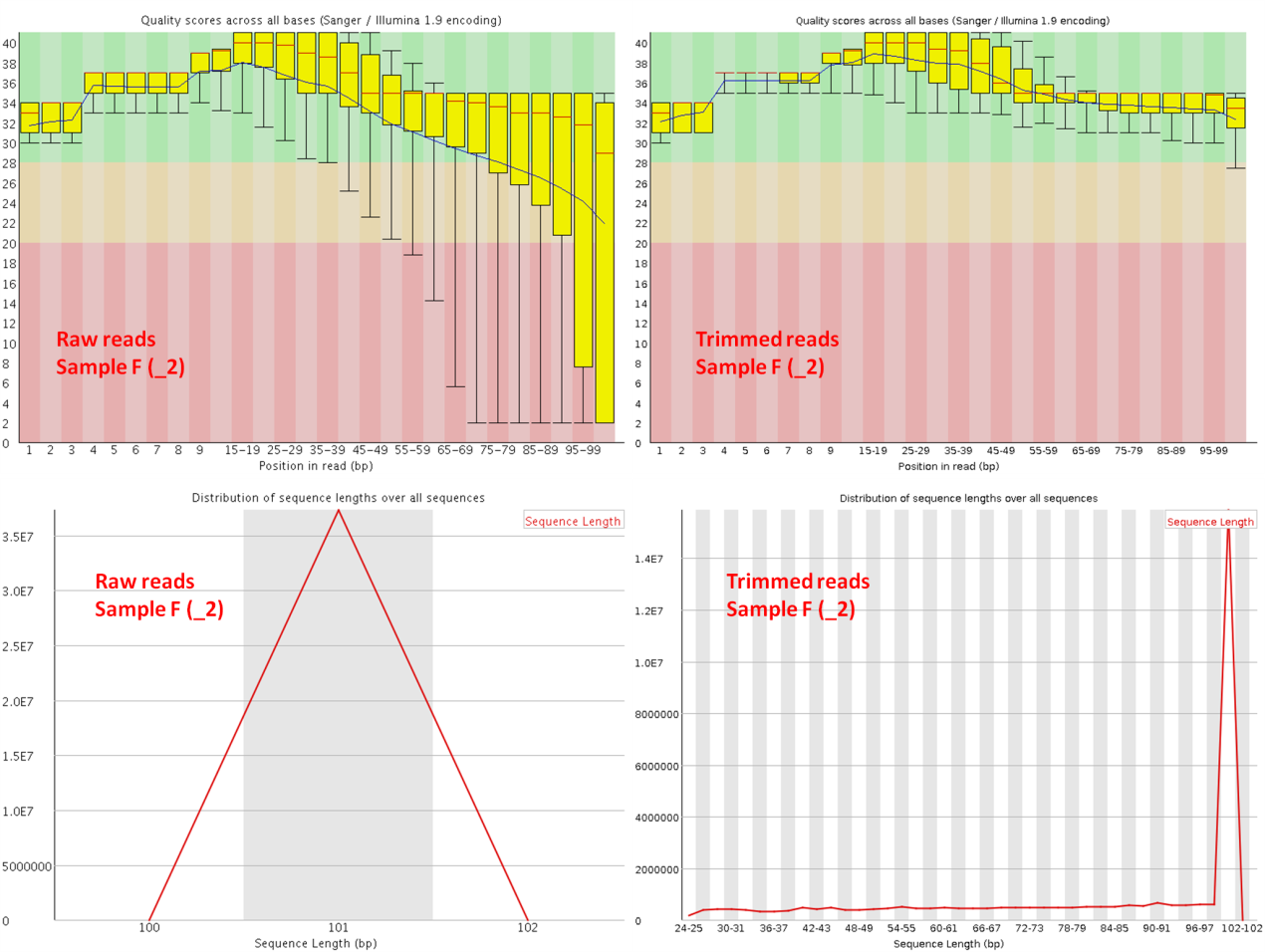

Supplement: Additional file 2: — Quality assessment of raw and trimmed dataset. (DOCX 7557 kb) [file 12866_2016_839_MOESM2_ESM.docx]
