# Supplementary material for: De novo transcriptomic analysis of Chlorella sorokiniana reveals differential genes expression in photosynthetic carbon fixation and lipid production
Source: BMC Microbiol. 2016 Sep 26;16:223. doi: 10.1186/s12866-016-0839-8 (PMC5037625; doi:10.1186/s12866-016-0839-8)
Supplement: Additional file 7: — Modified Kuhl medium. (DOCX 15 kb) [file 12866_2016_839_MOESM7_ESM.docx]

**Table S7 Modified Kuhl medium**

| components | mg·L^-1^ |
| --- | --- |
| NaH_2_PO_4_·2H_2_0 | 675 |
| Na_2_HPO_4_ | 71 |
| MgSO_4_·7H_2_O | 246.5 |
| EDTA·Na_2_ | 9.3 |
| H_3_BO_3_ | 0.061 |
| Cacl_2_·2H_2_O | 14.7 |
| FeSO_4_·7H_2_O | 6.95 |
| ZnSO_4_·7H_2_O | 0.287 |
| (NH_4_)Mo_7_O_24_·4H_2_O | 0.01235 |
| MnSO_4_·H_2_O | 0.169 |
| CuSO_4_·5H_2_O | 0.0249 |
| KNO_3_ | 0.8% (nitrogen-replete) |
|  | 0.2% (nitrogen-limited) |
|  | 0.033% ([photoautotrophy](app:ds:photoautotrophy)) |
| Glucose | 4% (heterotrophy) |
|  | No supply ([photoautotrophy](app:ds:photoautotrophy)) |
