# Supplementary material for: De novo transcriptomic analysis of Chlorella sorokiniana reveals differential genes expression in photosynthetic carbon fixation and lipid production
Source: BMC Microbiol. 2016 Sep 26;16:223. doi: 10.1186/s12866-016-0839-8 (PMC5037625; doi:10.1186/s12866-016-0839-8)
Supplement: Additional file 8: — Python scripts. (DOCX 18 kb) [file 12866_2016_839_MOESM8_ESM.docx]

In order to filter bases that are unsuitable for the following analysis, we wrote three Python scripts to preprocess the raw reads, whose source codes are as follows with the necessary description of the functions and usages. And these three Python scripts should be used in the following order, namely low_quality_base_trimmer.py, N_trimmer.py, and short_read_trimmer.py at last.

**The source code of the first Python script: low_quality_base_trimmer.py.**

#!/bin/python

# Program: low_quality_base_trimmer.py

# ------------------------------

# This program filters sequences base on quality,and keeps continuous sequence with higher quality score than the given quality threshold.

#

# Input: The fq file and quality threshold

#

# Output: The fq file with cotinuous sequence among which each base's quanlity score is higher than than the quality threshold given

#

# Example: python low_quality_base_trimmer.py 2_48h_1.fq 25

#

#

import sys

def low_quality_base_trimmer(fq,quality_threshold):

flag = 0

low_quality_base_num = 0

f = open(fq,'r')

f_trimmer = open(fq.rstrip('.fq')+'_low_quality_base_trimmer.fq','w')

for line in f:

i = 0

fragment_sequence = []

fragment_qualityscore = []

firstline = line

sequence = f.next()

thirdline = f.next()

qualityscore= f.next()

if len(sequence) != len(qualityscore):

sys.stderr().write('the length of sequence is not equal to the length of quality score!!!\n')

return flag

while '!' <= qualityscore[i] < chr(int(quality_threshold)+33) and qualityscore[i] != '\n':

i += 1

low_quality_base_num += 1

if qualityscore[i] == '\n':

continue

elif chr(int(quality_threshold)+33) <= qualityscore[i] <= 'J':

for j in range(i+1,len(qualityscore)):

if chr(int(quality_threshold)+33) <= qualityscore[j] <= 'J' and qualityscore[j] != '\n':

continue

elif qualityscore[j] == '\n':

fragment_qualityscore.append(qualityscore[i:j])

fragment_sequence.append(sequence[i:j])

break

elif '!' <= qualityscore[j] < chr(int(quality_threshold)+33):

fragment_qualityscore.append(qualityscore[i:j])

fragment_sequence.append(sequence[i:j])

i = j+1

low_quality_base_num += 1

else:

sys.stderr.write('the quality score was out of range!!\n')

return flag

if len(fragment_qualityscore) != len(fragment_sequence):

sys.stderr.write('there is not one-to-one correspondence between the sequence and the quality score!!!')

return flag

max_len_qualityscore = fragment_qualityscore[0]

max_len_index = 0

for k in range(0,len(fragment_sequence)):

if len(fragment_qualityscore[k]) > len(max_len_qualityscore):

max_len_qualityscore = fragment_qualityscore[k]

max_len_index = k

else:

continue

max_len_sequence = fragment_sequence[max_len_index]

if len(max_len_sequence) != len(max_len_qualityscore):

sys.stderr.write('error, line 68.')

return flag

f_trimmer.write(firstline)

f_trimmer.write(max_len_sequence+'\n')

f_trimmer.write(thirdline)

f_trimmer.write(max_len_qualityscore+'\n')

f.close()

f_trimmer.close()

print(fq+'\nquality threshold %d\nlow quality base number %d' %(int(quality_threshold),low_quality_base_num))

flag = 1

return flag

if __name__ == '__main__':

if low_quality_base_trimmer(sys.argv[1],sys.argv[2]):

print 'the function low_quality_base_trimmer.py has done!\nthe result was in file "***_low_quality_base_trimmer.fq"'

else:

print 'MUST BE ERROR!!!'

**The source code of the second Python script: N_trimmer.py.**

#!/bin/python

# Program: N_trimmer.py

# ------------------------------

# This program trims dubious base 'N' and keeps continuous sequence with higher quality score than the given quality threshold.

#

# Input: The fq file wich has been processed by the script low_quality_base_trimmer.py

#

# Output: The fq file with cotinuous sequence among which there is no dubious base 'N'

#

# Example: python low_quality_base_trimmer.py 2_48h_1.fq

#

#

import sys

def N_trimmer(fq):

flag = 0

N_num = 0

f = open(fq,'r')

f_trimmer = open(fq.rstrip('.fq')+'_N_trimmer.fq','w')

for line in f:

i = 0

fragment_sequence = []

fragment_qualityscore = []

firstline = line

sequence = f.next()

thirdline = f.next()

qualityscore = f.next()

if len(sequence) != len(qualityscore):

print 'the length of sequence is not equal to the length of qualityscore!'

return flag

while sequence[i] == 'N' and sequence[i] != '\n':

i += 1

N_num += 1

if sequence[i] == '\n':

continue

elif sequence[i] != 'N':

for j in range(i+1,len(sequence)):

if sequence[j] != 'N' and sequence[j] != '\n':

continue

elif sequence[j] == '\n':

fragment_sequence.append(sequence[i:j])

fragment_qualityscore.append(qualityscore[i:j])

break

elif sequence[j] == 'N':

fragment_sequence.append(sequence[i:j])

fragment_qualityscore.append(qualityscore[i:j])

i = j+1

N_num += 1

else:

print 'there are something wrong!'

return flag

if len(fragment_sequence) != len(fragment_qualityscore):

print 'the number of fragment_sequence is not one-to-one conrrespondce to the number of fragment_qualityscore!'

return flag

else:

max_len_sequence = fragment_sequence[0]

max_len_index = 0

for k in range(0,len(fragment_sequence)):

if len(fragment_sequence[k]) > len(max_len_sequence):

max_len_sequence = fragment_sequence[k]

max_len_index = k

else:

continue

max_len_qualityscore = fragment_qualityscore[max_len_index]

if len(max_len_sequence) != len(max_len_qualityscore):

sys.stderr.write('error, line 68.')

return flag

f_trimmer.write(firstline)

f_trimmer.write(max_len_sequence+'\n')

f_trimmer.write(thirdline)

f_trimmer.write(max_len_qualityscore+'\n')

print(fq+'\nN number %d' % N_num)

f.close()

f_trimmer.close()

flag = 1

return flag

if __name__ == '__main__':

if N_trimmer(sys.argv[1]):

print 'the function N_trimmer.py has done!!!\nthe result was in file "***_N_trimmer.fq"'

else:

print 'MUST BE REEOR!!!'

**The source code of the third Python script: short_read_trimmer.py.**

#!/bin/python

# Program: short_read_trimmer.py

# ------------------------------

# This program filters the reads based on length and remain the reads which is longer than the length threshold.

#

# Input: The fq file wich has been processed by the scripts low_quality_base_trimmer.py and N_trimmer.py

#

# Output: The fq file with the length of reads longer than the length threshold given

#

# Example: python low_quality_base_trimmer.py 2_48h_1.fq 25

#

#

import sys

def short_read_trimmer(fq,len_threshold):

flag = 0

read_num = 0

long_read_num = 0

short_read_num = 0

f = open(fq,'r')

f_trimmer = open(fq.rstrip('.fq')+'_short_read_trimmer.fq','w')

report = open(fq.rstrip('.fq')+'_short_read_trimmer_report','w')

for line in f:

firstline = line

sequence = f.next()

thirdline = f.next()

qualityscore = f.next()

if len(sequence) > 0 and sequence[0] != '\n':

read_num += 1

if len(sequence) >= int(len_threshold)+1:

f_trimmer.write(line)

f_trimmer.write(sequence)

f_trimmer.write(thirdline)

f_trimmer.write(qualityscore)

long_read_num += 1

else:

short_read_num += 1

else:

print 'some reads have no any base!!!'

return flag

report.write('************************************************************************\n')

report.write(fq+'\nLength threshold %d\nTotal reads number %d\nLong reads number %d\nShort reads number %d\n' % (int(len_threshold),read_num,long_read_num,short_read_num))

f.close()

f_trimmer.close()

report.close()

print 'total reads number %d\nlong_read_number %d\nshort_read_number %d' % (read_num,long_read_num,short_read_num)

flag = 1

return flag

if __name__ == '__main__':

if short_read_trimmer(sys.argv[1],sys.argv[2]):

print 'The function short_read_trimmer.py has done!!!\nThe result was in file "***_short_read_trimmer.fq"\nThe report was in file "***_short_read_trimmer_report"'

else:

print 'MUST BE ERROR!!!'
